# The Impact of Prenatal Exposure to Dexamethasone on Gastrointestinal Function in Rats

**DOI:** 10.1371/journal.pone.0161750

**Published:** 2016-09-01

**Authors:** Fátima Ramalhosa, Carina Soares-Cunha, Rui Miguel Seixal, Nuno Sousa, Ana Franky Carvalho

**Affiliations:** 1 Life and Health Sciences Research Institute, School of Health Sciences, University of Minho, Braga, Portugal; 2 Life and Health Sciences Research Institute/Biomaterials, Biodegradables and Biometrics Associate Laboratory, Braga/Guimarães, Portugal; 3 General Surgery Department, Hospital of Braga, Braga, Portugal; University of California Los Angeles, UNITED STATES

## Abstract

Antenatal treatment with synthetic glucocorticoids is commonly used in pregnant women at risk of preterm delivery to accelerate tissue maturation. Exposure to glucocorticoids during development has been hypothesized to underlie different functional gastrointestinal (GI) and motility disorders. Herein, we investigated the impact of *in utero* exposure to synthetic glucocorticoids (*iuGC*) on GI function of adult rats. Wistar male rats, born from pregnant dams treated with dexamethasone (DEX), were studied at different ages. Length, histologic analysis, proliferation and apoptosis assays, GI transit, permeability and serotonin (5-HT) content of GI tract were measured. *iuGC* treatment decreased small intestine size and decreased gut transit. However, *iuGC* had no impact on intestinal permeability. *iuGC* differentially impacts the structure and function of the GI tract, which leads to long-lasting alterations in the small intestine that may predispose subjects prone to disorders of the GI tract.

## Introduction

The intrauterine environment is crucial for normal structural and functional development. Exposure to prenatal adverse events cause persistent alterations over the lifespan [1]. Glucocorticoids (GC) are prescribed in obstetric and paediatric pathologies, in particular to pregnant women at risk of preterm delivery. Approximately 7% of pregnant women in Europe and North America are treated with dexamethasone (DEX) or betamethasone to promote lung maturation in foetuses [2]. GC are secreted in the adrenal cortex, under the control of the hypothalamic-pituitary-adrenal (HPA) axis. A balanced HPA axis activity is required for normal foetuses development, since endogenous corticosteroids are essential for normal growth and organogenesis during gestation [3]. The impact of *in utero* exposure to synthetic glucocorticoids (*iuGC*), on the brain, has been studied extensively in the past few decades. Changes in behaviour, impairment in working memory and attention deficits have been described in children born from mothers treated with synthetic GC during pregnancy [4–6]. In animal studies, *iuGC* has been shown to induce anxiety in the offspring and predispose them to depressive-like behaviour [7]. Importantly, the period of injection of DEX has been shown to be critical for the long-lasting effects on behaviour in adulthood [8,9].

It is noteworthy that *iuGC* has been shown to disrupt the HPA axis [7–10]. Importantly, the dysregulation of the HPA axis has been associated with different functional gastrointestinal (GI) and motility disorders, such as Irritable Bowel Syndrome (IBS) [11–12]. IBS is characterized by chronic abdominal pain and altered bowel habits, associated with stress-related psychiatric disorders. It is the most common functional GI disorder, affecting 7–10% of the general population [13]. Early adverse life events (EALs) have also been linked to IBS development in adulthood [14, 15]. Accordingly, neonatal maternal separation (MS) in rodents induces IBS-like features, such as visceral hyperalgesia, anxiety and gut dysmotility [16–23]. These features are more evident after exposure to chronic stress, suggesting maladaptation to stress, as is seen in IBS patients [16–20]. It is clear that EALs are associated with the development of psychiatric disorders and functional GI alterations, but the impact of adverse events during gestation on GI function remains unclear.

Using the *iuGC* animal model, which mimics the therapy given in pregnant women at risk of preterm delivery, we intend to explore the impact of prenatal administration of DEX in GI function.

## Materials and Methods

### Animals and prenatal treatments

All animal experiments were performed in accordance with National and European Commission guidelines for the care and handling of laboratory animals (European Union Directive 2010/63/EU) and were approved by the National Veterinary Directorate (DGV-023432) and by the local Animal Ethical Committee (Subcomissão de ética em ciências da Vida e Saúde of the Minho University Ethics committee). Female Wistar rats (~150–200 g) were obtained from Charles River Laboratories (Barcelona, Spain). All animals were housed in an animal facility at 22°C, relative humidity of 55%, in a 12 h light and 12 h dark cycle, with food and water available *ad libitum* (diet 4RF21, Mucedola, Settimo Milanese, Italy). 2 or 3 females were housed with a male and, in the day sperm was seen in a vaginal smear, was designated as day 1 of pregnancy. Pregnant females were housed individually and randomly assigned to the treatment group *iuGC* or Control. Dams were injected with DEX (1 mg/kg/day in 4% ethanol/sesame oil, 1 mg/mL; Sigma-Aldrich, Saint Louis, MO, USA) or sesame oil (Sigma-Aldrich, Saint Louis, MO, USA) subcutaneously on days 18 and 19 of pregnancy [7]. Few studies exist comparing cortisol and GR affinity between rodents and humans; it is suggested that guinea pig GR has 4-fold lower affinity for synthetic GC (sGC) than human GR [24]. Thus, the dosage used in this study (1mg/mL) has been found to be comparable to the dose used in pregnant women (0.3–0.5 mg/kg) [25]. Weaning occurred at postnatal day 21 and male rats were housed 2 animals per cage, according to prenatal treatment (Control or *iuGC*). The offspring males, from each dam were, divided in four groups in order to use the same birth colony at different ages: 24 hours and 1, 3 and 8 months old. We used 8 control and 10 *iuGC* damns. Both control and *iuGC* had an average litter size of 8 with approximately 60% of males and 40% of females.). Each time point represent different important human phases: third trimester of gestation, preadolescence, beginning of adulthood and mature adulthood [26].

### Neonatal assessment

After spontaneous delivery at term (22 d), 2 new-borns male rats from each litter, were sacrificed by decapitation. The abdominal cavity was opened and, after removing the small intestine, the length was measured.

### Handling and oral gavage

Prior to habituation to oral gavage, all animals were handled daily for 5 minutes, over one week. Habituation to oral gavage took 5 days. On the 4^th^ day animals received Fluorescein isothiocyanate-conjugated (FITC) dextran 70 kDa (FD-70) (Sigma-Aldrich, Saint Louis, MO, USA), to measure permeability; and, on the last day, animals received Carmine red (Sigma-Aldrich, Saint Louis, MO, USA) one hour before sacrifice, to measure gut transit. A curved-stainless steel feeding needle with different lengths: 50 nm for 1 month old and 100 mm for 3 and 8 months old (Fine Science Tools, Heidelberg, Germany) was used to perform the gavage technique. Animals were sacrificed by 1 mL intraperitoneally (*i*.*p*.) injection of a lethal dose of pentobarbital (Eutasil 200mg/mL, Algés, Portugal).

### Gastrointestinal permeability

*In vivo* GI permeability was measured one day before sacrifice. Rats were administered0.2 mL FD-70, by oral gavage. FD-70 was dissolved in phosphate-buffered saline (PBS, pH 7.4) to reach a concentration of50 mg/mL (Sigma-Aldrich, Saint Louis, MO, USA) [27]. One hour after administration, venous blood was collected by tail vein puncture. Preliminary data showed that at 1 hour most of carmine dye has not yet reached the colon. Thus, at this time point we will be assessing small intestine function. Blood samples were centrifuged (10.000 rpm at 4°C) for 5 minutes. Plasma (50μl) was mixed with an equal volume of PBS (pH 7.4) and added to a 96-well microplate. The concentration of fluorescein was determined by spectrophotometry (Model 680 microplate reader, Bio-rad, USA) with an excitation wavelength of 485 nm an emission wavelength of 530 nm, using a calibration curve.

### Gastrointestinal Transit

Carmine red, which cannot be absorbed from the lumen of the gut, was used to study *ex vivo* GI transit [28]. On the 5^th^ day of gavage habituation, rats were given 0.2 mL of Carmine red dye (Sigma-Aldrich, Saint Louis, MO, USA) suspended at the concentration of 6% (w/v) in distilled water containing 0.5% methylcellulose (Davilose forte, Barcarena, Portugal) [29]. One hour after carmine administration, rats were sacrificed and transcardially perfused with 50 mL saline (0.9% w/v of NaCl). The stomach, small and large intestine were dissected and the distance travelled by Carmine red in the small intestine was measured. GI transit was assessed as the ratio between the distance travelled by the Carmine red and the total length of the small intestine.

### Morphological analysis in the Small Intestine

The abdominal cavity was opened and stomach, small intestine and colon were removed. Since GI tract is easily over-stretched, we placed the whole gut in a sterile workbench and the length was measured with a ruler. For stomach we measured the biggest diameter (between pyloric antrum and fundus) and whole small intestine (duodenum, jejunum and ileum) was considered. All measurements were performed by a blinded investigator. After dissection, the same 2–3 cm segment distal ileum of each animal was kept for 24 h in 3.7% paraformaldehyde (PFA) solution. Then, tissues were removed from PFA and embedded in paraffin blocks, sectioned, placed on glass microscope slides and stained with haematoxylin and eosin. Smooth-muscle and submucosa plus mucosa morphometric were examined by light microscopy at 4 x magnification using an Olympus BX51 (Olympus, Lisbon, Portugal) and analysed with Visiopharm Integrator System 2.12.3.0 Software (Visiopharm, Broomfield, CO). The thickness values of submucosa plus mucosa and muscular layers are an average of 3 different measurements, in transversal tissue sections, in a blind fashion.

### Proliferation and apoptosis analysis in small intestine

#### Proliferation Assay

For the proliferation studies, animals were injected intraperitoneally with bromodeoxyuridine (BrdU, 50 mg/Kg, Sigma-Aldrich, Saint Louis, MO, USA) one hour before sacrifice, after the last gavage. BrdU is a synthetic nucleoside that is an analogue of thymidine and can be incorporated into the newly synthesized deoxyribonucleic acid (DNA) of replicating cells, substituting to thymidine during DNA replication. BrdU incorporation into DNA can be identified by immunostaining and reveals cells that were actively replicating their DNA. Paraffin-embedded tissue were dewaxed and permeabilized with Tris-buffered saline (TBS)/0.2% Triton X-100 (Sigma-Aldrich, Saint Louis, MO, USA) during 10 minutes. Antigen retrieval was performed during 20 minutes at lower power heating on the microwave with citrate buffer (2.282 g/L citric acid monohydrate, Sigma-Aldrich, Saint Louis, MO, USA, pH = 6.0), pre-heated at maximum power during one minute. Acidification was performed at room temperature with hydrochloride acid 2M during 30 minutes, endogenous peroxidase was inactivated with 9% H_2_O_2_ diluted in TBS in a 1:10 ratio during 15 minutes and non-specific protein bindings was blocked with 10% Bovine Serum Albumin (Sigma-Aldrich, Saint Louis, MO, USA) in TBS during 30 minutes. These procedures were alternated with a 3-minute washing with TBS 3 times each. The sections were exposed to monoclonal rat anti-BrdU antibody (1:100 diluted in TBS, Monoclonal Rat Anti-Bromodeoxyuridine Mo744, Clone BU20A, Dako, Lisboa, Portugal) overnight at 4°C and, the following day, they were incubated with avidin-biotin-peroxidase complex (UltraVision Large Volume Detection System Anti-Polivalent, HRP, ThermoScientific, Waltham, MA, USA) for 60 minutes at room temperature. Immunoreactivity was visualized with a revelation kit (ImmPACT NovaRED Peroxidase Substract, SK-4805, Vector Laboratories, Burlingame, CA, USA) according to manufacturer’s protocol. All sections were subsequently counterstained with 50% haematoxylin and eosin using an automatic stainer (Leica Autostainer XL, Leica, Wetzlar, Germany). As a negative control one of the sections was not incubated with the primary antibody, which did not results in any staining of the tissue.

#### Apoptosis Assay

*In situ* cell death detection kit (TUNEL assay, Roche Diagnostics, Amadora, Portugal) labels DNA strand breaks generated during apoptosis, which allow us to stain apoptotic cells. Paraffin-embedded tissue were deparaffinised, incubated for 2 min on ice with Phosphate buffered saline (PBS)/0.1% Triton X-100 (Sigma-Aldrich, Saint Louis, MO, USA), immersed in citrate buffer (0.01 M, pH = 6.0, Sigma-Aldrich, Saint Louis, MO, USA) for 1 minute at 750 W and rapidly cooled in distilled water. The slides were treated for 15 min at room temperature with proteinase K (20 μg mL^-1^ in 10 mM Tris/HCl, pH 7.6) and non-specific protein bindings was blocked with 3% Bovine Serum Albumin (Sigma-Aldrich, Saint Louis, MO, USA) in PBS during 30 minutes. No inhibition of endogenous peroxidase was performed because hydrogen peroxidase (H_2_O_2_) pre-treatment has been reported to weaken terminal deoxynucleiotidyl transferase (TdT) activity and induce DNA breaks [30]. Subsequently incubated with the TUNEL reaction mixture (Label solution and enzyme solution) for 60 min at 37°C. For positive control, DNA strand breaks were induced in all nuclei by pre-incubation of the sections for 30 min at 37°C with 1U/ml DNAse I (Roche Diagnostics, Amadora, Portugal) in 10 mM Tris-HCl buffer (pH 7.5, to which 1 mM MgCl_2_ and 0.1% BSA were added). For negative control, the section is incubated with the label solution, instead of TUNEL reaction mixture. The slides were again immersed in 3% BSA (Sigma-Aldrich, Saint Louis, MO, USA) and incubated with Converter-POD (Anti-fluorescein antibody, Fab fragment from sheep, conjugated with horse-radish peroxidase) for 30 min at 37°C in a humidified chamber, followed the revelation reaction (ImmPACT NovaRED Peroxidase Substract, SK-4805, Vector Laboratories, Burlingame, CA, USA). All sections were subsequently counterstained with 50% hematoxyline and eosin using an automatic stainer (Leica Autostainer XL, Leica, Wetzlar, Germany).

#### Proliferation and apoptosis quantification

The number of labelled cells was estimated using stereological method: mucosa plus submucosa and muscularis layers were selected and the software (Visiopharm Integrator System 2.12.3.0 Software, Visiopharm, Broomfield, CO) randomly selects 400μm^2^ probes from 10% of total epithelium area to make the counts. The size of area in each animal varies through 0.2 m^2^ and 0.4 m^2^, accordingly with the animal age. This was performed on Olympus BX51 (Olympus, Lisbon, Portugal) and images were recorded using Pixelink PL-A622-KIT (Pixelink, Ottawa, Ontario, Canada).

### Serotonin quantification

On the sacrifice day, 2–3 cm of the ileum were rapidly dissected, snap-frozen (dry ice) and stored at -80°C until use. Perchloic acid (0.2 N) was added to each sample and, after disruption and sonication (1 min on ice), samples were centrifuged at 4°C at 5000 rpm. The supernatant was filtered through a Spin-X high-performance liquid chromatography (HPLC) column (Costar, Lowell, MA, USA) to remove debris. Levels of 5-HT was measured by HPLC combined with electrochemical detection using a Gilson instrument (Gilson, Middleton, WI, USA), fitted with an analytical column (Supelco Supelcosil LC-18 3μM, flow rate: 1.0 ml/min, Bellefonte, PA, USA) as previously described [31]. Briefly, 150μl supernatant aliquots were injected into the system, using a mobile phase of 0.7 M aqueous potassium phosphate (pH 3.0) in 10% methanol, 1-heptanesulfonic acid (222mg l^-1^, Sigma-Aldrich, Saint Louis, MO, USA) and Na-EDTA (40 mg l^-1^, Sigma-Aldrich, Saint Louis, MO, USA). A standard curve using known concentrations of serotonin was run each time.

### Data analysis

Data are presented as mean value per group ± standard deviation (SD). Statistical analysis was performed using SPSS (IBM SPSS Statistics 22 software, New York, USA) and graphs were made in GraphPad Prism 6.0 (GraphPad Software, Inc., La Jolla, CA, USA). The mean values of multiple groups were determined by two-way factorial analysis of variance—ANOVA (age x group), followed by the Bonferroni’s *post hoc* multiple comparison test for group differences determination. Student’s t-test was used to compare the two groups at 24 h of life. Normality test (Kolmogorov-Smirnoff test) and equality of variances (Levene’s test) were evaluated before the statistical tests. The significance was set at *P* < 0.05.

## Results

### *iuGC* exposure decreases the length of the small intestine

**Fig 1:**
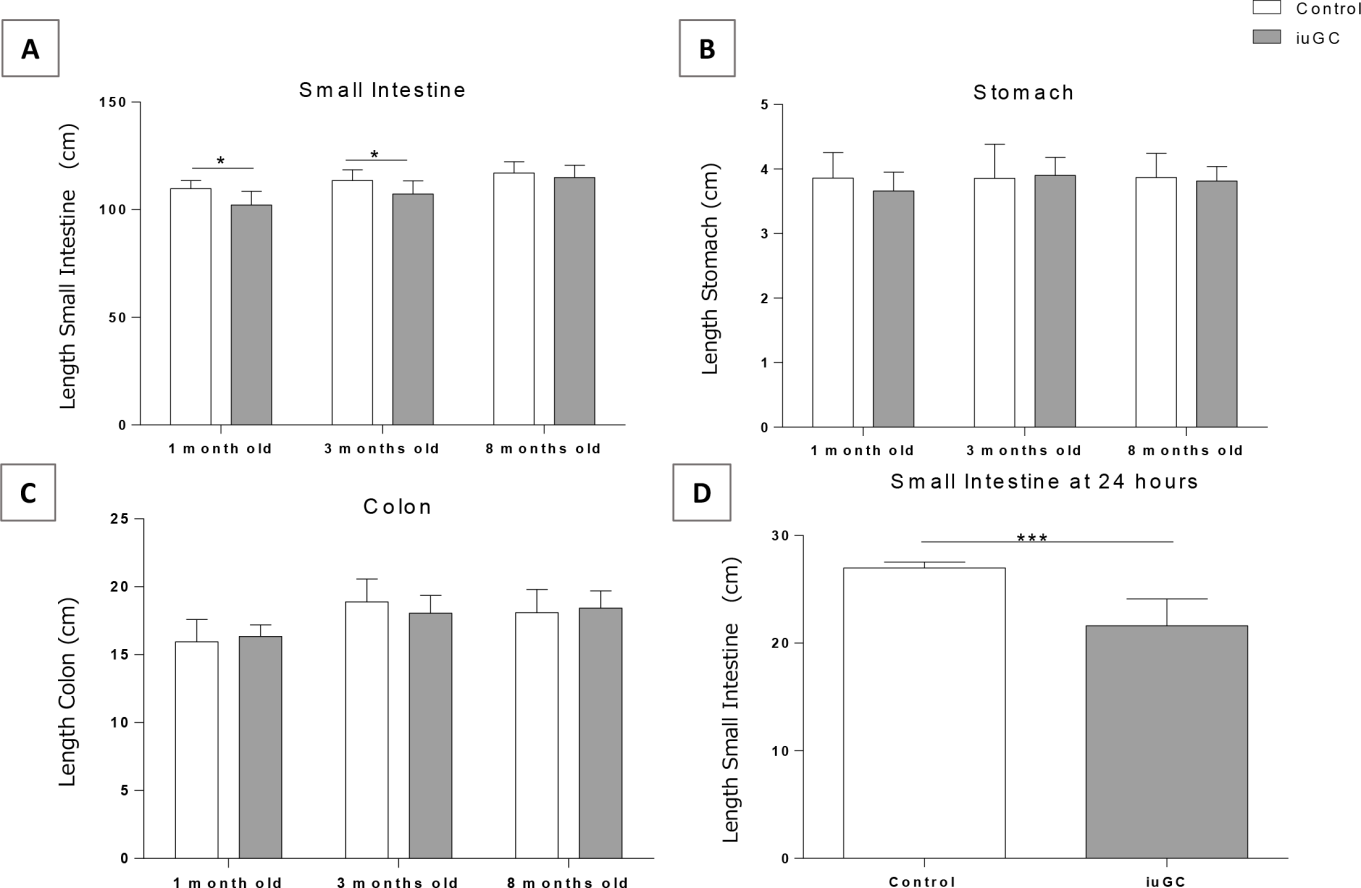
*In utero* glucocorticoid exposure decreases the length of small intestine at 24 hours, 1 and 3 months old. (A) The length of small intestine is shorter in *iuGC* rats at 1 and 3 months old, n = 6, 17, 6 CTR and n = 9, 10, 7 *iuGC*. (B) Stomach length in control and *iuGC* animals, n = 10, 9, 6 CTR and n = 10, 11, 7 *iuGC*. (C) The length of colon in control and *iuGC* animals, n = 10, 9, 6 CTR and n = 10, 11, 7 *iuGC*. (D) Small Intestine is shorter in *iuGC* animals at 24 hours of life, n = 4 CTR and n = 13 *iuGC*. *iuGC*, *in utero* glucocorticoid exposed animals. *P <0.05, *** *P* <0.001.

To evaluate the impact of prenatal GC exposure in GI structure, we measured the length of different GI organs (stomach, small intestine and colon). Two-way ANOVA revealed a significant influence of age (F_2,41_ = 12.015, *P* = 0.0001) and group (F_1,41_ = 10.658, *P* = 0.002) on length of small intestine. Compared with controls, *iuGC* animals presented shorter small intestine (Fig 1A) at 1 (109.75 ± 3.79 cm in CTR vs. 102.22 ± 6.34 cm in *iuGC*) and 3 months of age (113.22 ± 3.38 cm in CTR vs. 107.3 ± 6.15 cm in *iuGC*). No differences in size of the stomach and colon were detected between the CTR and the *iuGC* group at all ages (Fig 1B and 1C), suggesting a preferential and selective effect of *iuGC* in the small intestine. To evaluate whether this alteration is congenital or a postnatal adaptation, we assessed the length of small intestine in newborn rats at 24 h postnatal (Fig 1D). At this time, *iuGC* animals already showed a reduction in small intestine size (26.27 ± 0.96 cm in CTR vs. 21.62 ± 2.5 cm in *iuGC*). The length of the small intestine was normalized to the animal weight, and no statistical differences were found (S1 Fig)

### *iuGC* does not impact microscopic structure but affects proliferation and apoptosis rate

**Fig 2:**
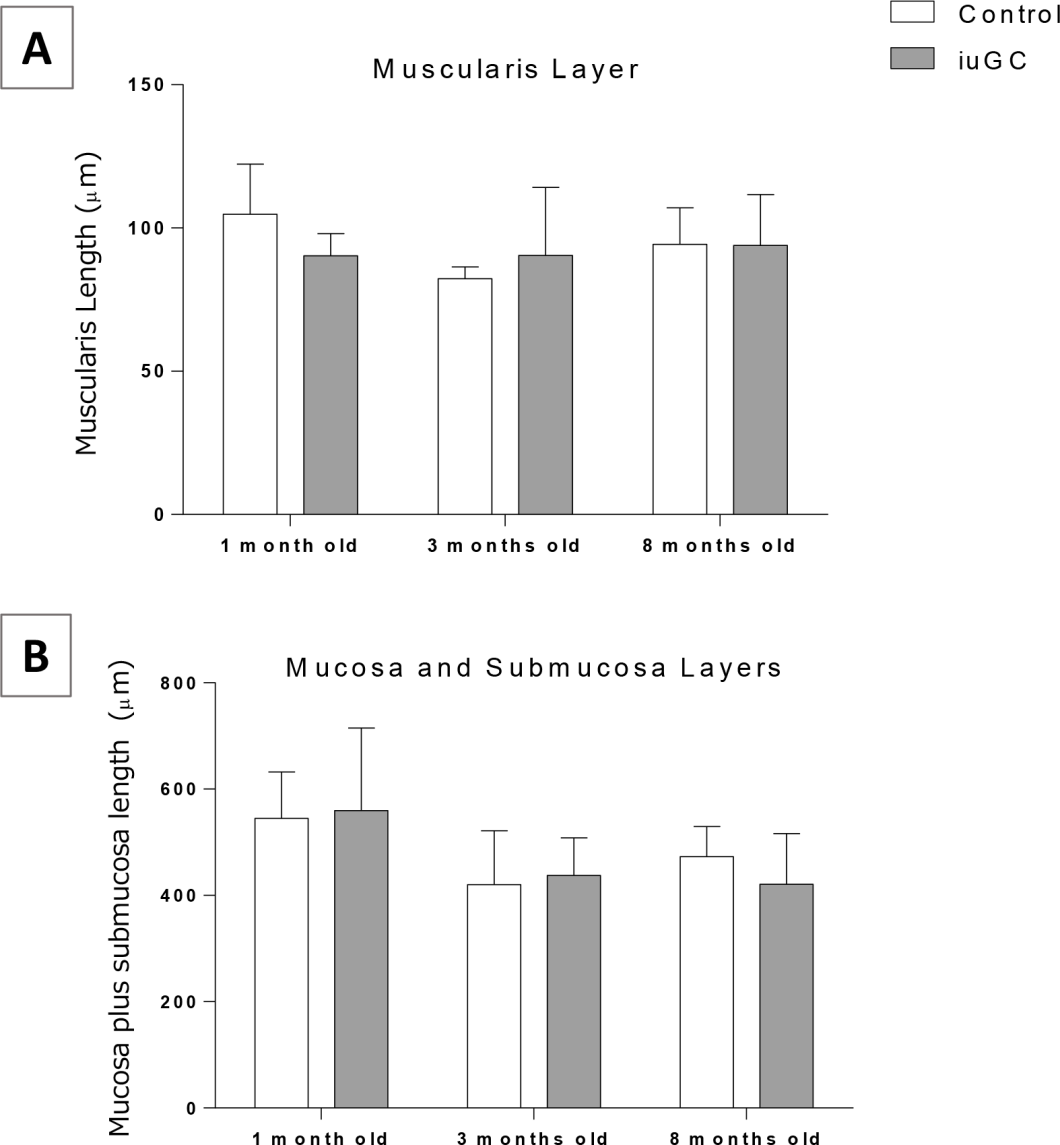
Muscularis and submucosa plus mucosa layers length in small intestine. (A) Muscularis and (B) submucosa plus mucosa layers at 1, 3 and 8 months old. There were no differences between groups. *iuGC*, *in utero* glucocorticoid exposed animals. (A) n = 6, 4, 6 CTR and n = 5, 10, 7 *iuGC*. (B) n = 6, 7, 6 CTR and n = 5, 9, 7 *iuGC*.

**Fig 3:**
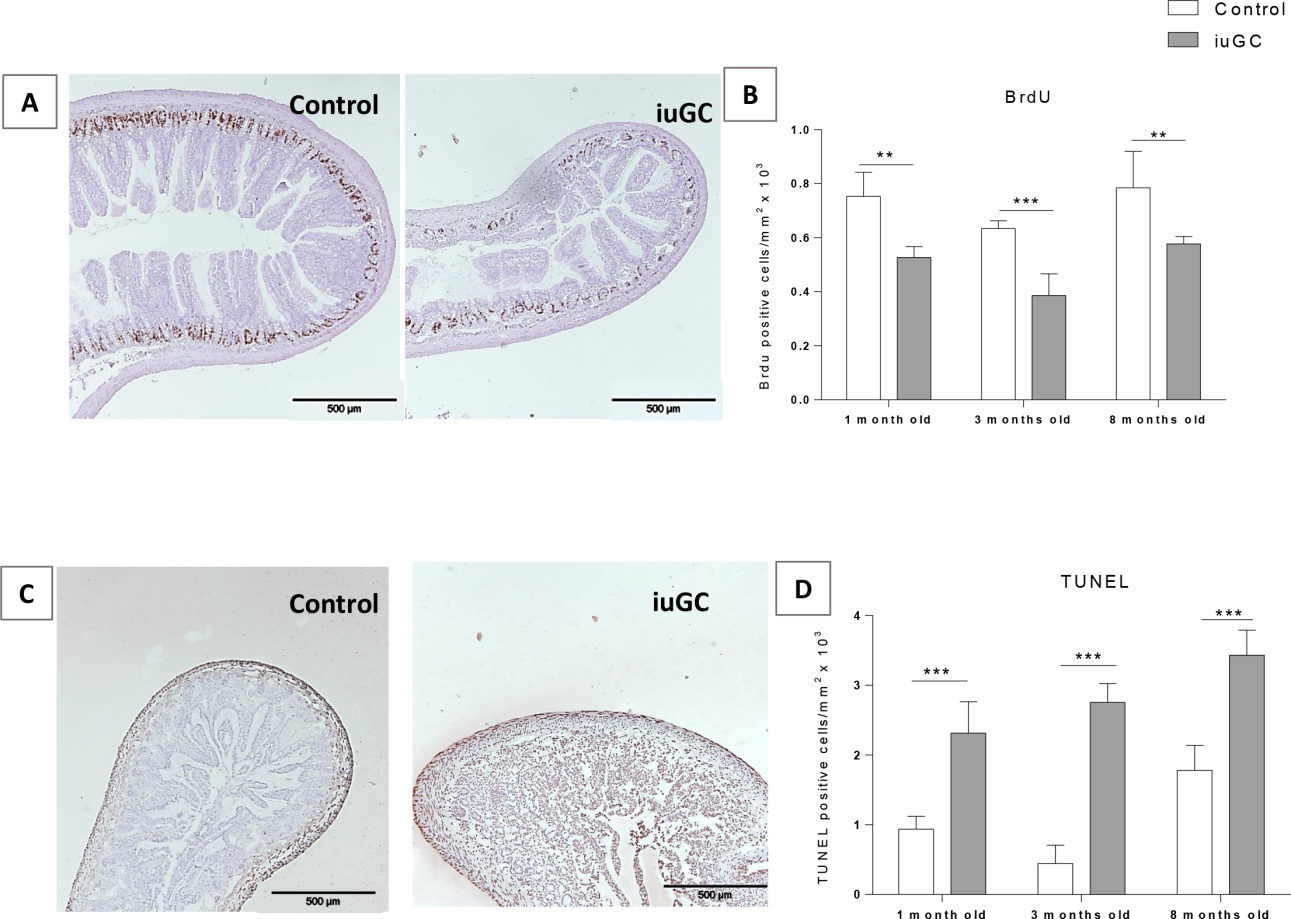
Prenatal treatment decreases proliferation and increases apoptosis in small intestine. (A) Proliferation was assessed by BrdU staining. (B) Proliferation rate is decreased within the crypts in *iuGC* rats, n = 3, 4, 5 CTR and n = 3, 6, 3 *iuGC*. (C) Apoptosis rate was assessed by TUNEL assay. (D) Apoptosis is increased in *iuGC* rats (mostly within the submucosa and mucosa layers), n = 6, 3, 5 CTR and n = 5, 3, 6 *iuGC*. *iuGC*, *in utero* glucocorticoid exposed animals.** *P* <0.01, *** *P* <0.001. Scale bars: 500 μm.

We further investigated the impact of *iuGC* on the morphology of the small intestine. At the structural level, we evaluated the thickness of small intestine wall and no differences were found in the thickness of muscular and submucosal plus mucosal layers (Fig 2). To clarify the decreased in total length of small intestine, despite normal microscopic structure, we performed proliferation and apoptosis assays. As shown in Fig 3A, BrdU positive cells were found almost exclusively within epithelium crypts, where proliferation is known to be higher [32–34]. Two-way ANOVA revealed an effect of age (F_2, 18_ = 9.625; *P* = 0.001) and group (F_1, 18_ = 40.54; *P* = 0.0005) on proliferation. Decreased proliferation was seen in *iuGC* animals at all ages (Fig 3B).

Regarding apoptosis, a TUNEL assay was used to detect DNA fragmentation indicative of apoptosis. An interaction age x group (F_2, 22_ = 3.828; *P* = 0.037), age (F_2, 22_ = 29.621; *P* = 0.0005) and group (F_1, 22_ = 20.341; *P* = 0.0001) was found. *iuGC* animals showed increased apoptosis at all time-points (Fig 3D). In control animals apoptosis was seen mostly within the muscular layer, while in *iuGC* animals there were high levels of apoptosis also within the submucosa and mucosa layers (Fig 3C). Despite of short small intestine and dysregulation of proliferation/apoptosis rates, we did not found alterations on layer’s length, which could mean that *iuGC* impacts on a particular cell subpopulation.

### *iuGC* animals present dysmotility despite normal intestinal permeability

**Fig 4:**
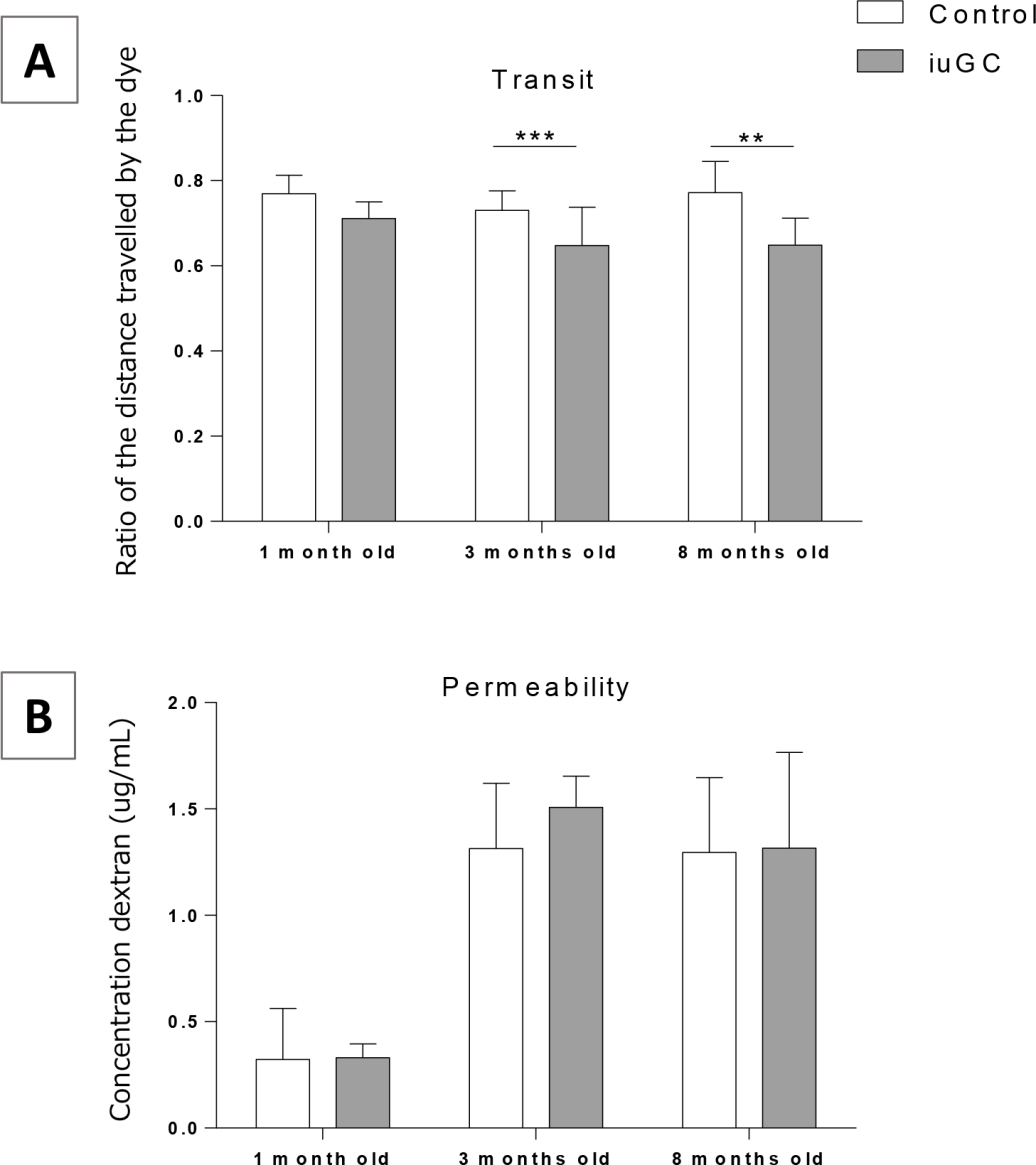
*iuGC* animals at 3 and 8 months old have a decreased GI transit. **No differences in intestinal permeability.** (A) Ratio of the distance travelled by red dye and total small intestine length. Ratio is lower at 3 and 8 months old. There was no differences among groups at 1 month old (*P* = 0.07), n = 7, 17, 6 CTR and n = 6, 19, 7 *iuGC*. (B) Intestinal permeability in Control and *iuGC* animals at 1, 3 and 8 months old. There were no differences between groups, n = 3, 9, 5 CTR and n = 4, 10, 7 *iuGC*. *iuGC*, *in utero* glucocorticoid exposed animals. ** *P* <0.01, *** *P* <0.001.

To assess the impact of *iuGC* on GI function we evaluated GI permeability and transit. To assess permeability and transit, animals were given (by oral gavage) FITC-dextran and Carmine red, respectively. As oral gavage can be a stressor and may alter gut transit baseline [35, 36], we performed a preliminary study to evaluate the impact of oral gavage in GI transit. Animals showed decrease gut transit when given acute oral gavage when comparing to repeated gavages (habituation) (S2 Fig) suggesting that a single exposure to oral gavage functions as an acute stressor. Thus, in our studies, all animals had a period of habituation to oral gavage (5 days). After this period, a red dye was given by oral gavage into the stomach. One hour after, the animals were sacrificed and the distance travelled by the red dye was measured. There is an effect of age (F_2, 52_ = 3.221; *P*<0.05) and group (F_1, 52_ = 20.163; *P*<0.001) on transit. *iuGC* animals at 3 and 8 months old had decreased GI transit (ratio between the distance travelled by the red dye and total length of small intestine) when compared to control animals (Fig 4A). At 1 month old, transit showed a tendency to be slower in *iuGC* rats (*P* = 0.07). Regarding permeability, we have measured *in vivo* intestinal permeability by luminal mucosa-to-blood flux of FITC-dextran. No changes were found in blood concentrations of FITC-dextran between animals, which is consistent with a normal functional intestinal barrier in *iuGC* animals (Fig 4B).

### *iuGC* animals have decreased 5-HT at 3 months old

**Fig 5:**
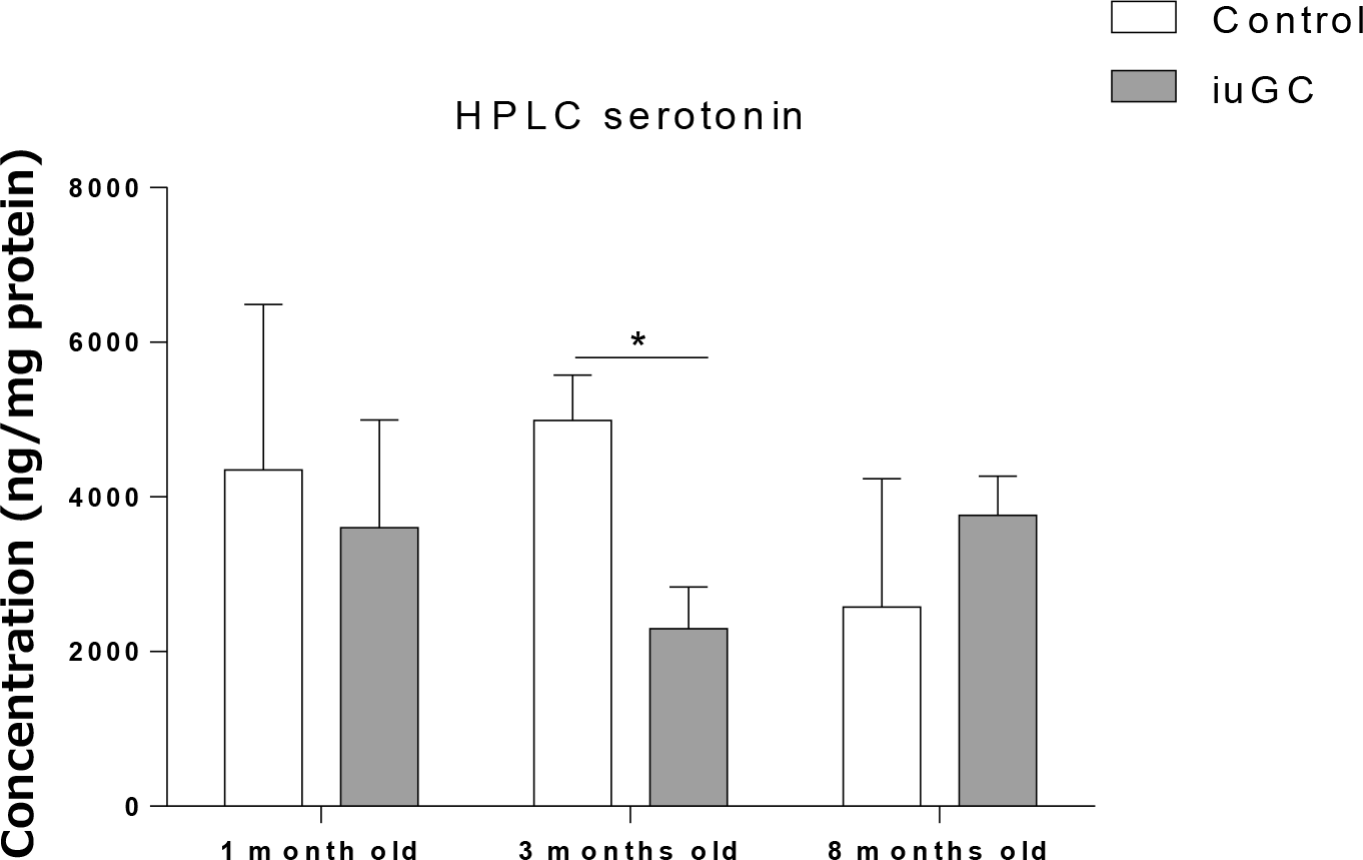
Serotonin levels, in small intestine, are decreased in *iuGC* animals at 3 months old. Serotonin levels are decreased in *iuGC* rats at 3 months old. There were no differences among groups at 1 and 8 months old, n = 10, 3, 4 CTR and n = 7, 5, 4 *iuGC*. *iuGC*, *in utero* glucocorticoid exposed animals.* *P* <0.05.

Most of 5-HT in the blood is derived from the GI tract and it has a crucial role in gut motility [37]. Thus, we quantified 5-HT levels in the small intestine. *iuGC* animals showed decreased levels of 5-HT at 3 months old (*P<*0.05) (Fig 5).

## Discussion

The present study shows the impact of prenatal administration of DEX on the GI tract of rats through gestation to young adulthood. *iuGC* differentially impacted gut size, leading to a reduction in small intestine total length without affecting the size of other GI organs, such as the stomach and the colon. Dysregulation of proliferation and apoptosis rates found in *iuGC* animal model could account for alterations on the normal ENS maturation and, consequently, GI motility. Moreover, it is shown that *iuGC* decreases gut transit without affecting intestinal permeability. *iuGC* decreases 5-HT levels in the small intestine at 3 months old, which may be related with the central serotonergic unbalance, which reinforce the importance of brain-gut axis.

Both DEX and betamethasone can be used as prevention of respiratory distress syndrome, as primary cause of early neonatal mortality and disability. Treatment protocols do not show a preference for one in particular, since meta-analysis of different studies directly comparing DEX with betamethasone found no statistically significant differences [38, 39]. The impact of DEX, prenatally administrated in rodents, has been studied in more detail since it is associated with central nervous system (CNS) negative effects [7, 40, 41].

DEX is a sGC, which binds exclusively to glucocorticoid receptors (GR), while cortisol (CORT, corticosterone in rodents) binds to both receptors: GR and mineralocorticoid receptors (MR), which are expressed in the gut [42]. In basal conditions, CORT shows a higher affinity for MR, but with stress, when CORT secretion is elevated, GR also become activated [11]. The expression of GR and MR varies through the GI tract, accounting for the different roles of each GI organ. The duodenum and jejunum express lower levels of MR and higher levels of GR, compared with ileum and colon. Nonetheless, binding to GR in the ileum seems to be higher than in the duodenum and jejunum [42, 43]. In contrast, MR binding assays showed no differences in affinity throughout the gut despite different protein distribution [42]. The distinct distribution of GC receptors may explain the differential impact of DEX administration in gut size, with decreased length of small intestine, where GR receptors are highly expressed [42, 43]. The levels of sGC, during gestation, are important for differentiation and maturation of various systems, such as the CNS [44–46] and the GI tract [47, 48]. In fact, sGC are widely used to promote lung maturation in preterm delivery [49]. Women at risk of preterm delivery, between 26 and 35 weeks of gestation, are treated with DEX which, besides promoting lung maturation, seems to decrease the risk of intraventricular harmorrhage, necrotizing entercololitis and neonatal death [49], suggesting a boosted maturation of these systems by *iuGC*. Although sGC are important for gut development they may not be essential for it, indeed, Gartner et al showed that mice lacking GR presented with normal gut development when compared to wild-type littermates [47].

While no significant changes were found at the microscopic level (no changes in layers development), there was a reduced length of the small intestine. Small intestine length is shorter in *iuGC* at 24 hours, 1 and 3 months old, which equates to the period from the third trimester to the beginning of the adulthood in humans [26]. No changes were found at 8 months old rats. In contrast to our findings, Majumdar et al showed that chronic administration of hydrocortisone (10 days) to pregnant rats increases small intestine and pancreas size at birth, emphasizing the role of corticoids in gut development [48]. Majumdar et al used hydrocortisone, which is known to have equal affinity to GC and MR, while in the present study DEX was used, which binds exclusively to GR [50]. Despite that, DEX may impact the expression of growing factors in the GI tract, affecting its length. Newborn mice receiving DEX showed decreased expression of transforming growth factor (TGF) and epidermal growth factor (EGF) in GI smooth muscle [51]. EGF has been shown to increase GI length when given artificial formula to rat pups, while TGF has been associated with myogenic alterations, namely morphology and contractile activity [52, 53]. TGF is expressed in human and rat ileum smooth muscle and has been implicated in GI maturation after birth in rat, when circulating GC levels increase (third week) [53–55]. On the other hand, TGF has no impact in small intestine size in adult animals [56]. At 8 months old, *iuGC* rats did not show differences in the small intestine size, which could reflect that the catch-up phenomenon seen in some preterm babies [57] may be delayed in the gut. Our results are in agreement with other studies, which show that *iuGC* delays puberty onset and neurodevelopment [10, 58].

In parallel, we found that *iuGC* decreased cell proliferation in the intestinal crypts and increased apoptosis, mostly in mucosa and submucosa layer. There is evidence supporting that DEX can induce apoptosis in different types of cells [44–46, 59–61]. In addition, there is also evidence that DEX can decrease gastric epithelium proliferation at the ulcer site [62]. In the ENS, as in the CNS, neurogenesis is essential after neuronal migration, but is through programed cell death (PCD) that regulated apoptosis provides an efficient control of population size and arrangement [63, 64]. In animals models, neurogenesis in the ENS has been found during gestation and adulthood, but the role of pre and postnatal PCD is not clear, since the majority of studies do not found apoptotic activity in the ENS [65–67]. However, recently, Wallace et al found PCD activity in pre-enteric neural crest cells (NCC) [68]. The mechanism by which cells undergo PCD following DEX administration are unclear. It would be interesting to better characterize these cells populations in further studies to better understand whether increased apoptosis is happening in glia or mast cells. Changes in glia cells could play as an opportunistic phagocyte, as it happens in the CNS, or dysfunction of the cells may induce loss of enteric neurons [69, 70]. The induction of apoptosis and the inhibition of proliferation by *iuGC* may affect the normal maturation process and organization of the ENS and produces long lasting alterations in cell cycle in the ENS.

The GI tract has its own nervous control through the ENS, which is capable of controlling all functions independently from CNS (but with possible modulation by the later). At E9.5-E10, neural crest cells (NCC) begin to migrate along the entire length of the GI tract, proliferating and differentiating into different enteric neurons and glia cells [71, 72]. At the same time, migration of smooth cells from mesoderm to the gut can be seen [73]. Spontaneous contractions of the duodenum and colon start at E13.5 and E14.5, as the circular muscle layer is already formed [73]. These contractions are dependent on muscular activity and are designated myogenic-mediated contractions. Organized contractions, responsible for the presence of motility patterns and mediated by neurons (neurogenic-mediated contractions), can be seen from E18.5 (duodenum) and 1 week after birth (colon) [74]. The delay between differentiation of neurons and the development of neurogenic-mediated motility patterns reflect the time needed for maturation of the complex connections within the ENS [74]. In the duodenum and jejunum, intracellular recordings show that slow waves are not present until E18.5 and E19, which coincides with the arise of KIT positive cells with the morphological features of myenteric interstitial cells of Cajal (ICC-MP) [74]. ICC-MP, derived from the mesenchyme, are seen as the pacemaker of the gut, being responsible for initiating gut contractions [75–78]. In this study, administration of DEX occurs on E18 and E19 of gestation, which matched the first neural mediated contractions and functional ICC-MP in the duodenum and jejunum of the small intestine. Thus, this animal model is probably not affecting the normal differentiation and migration of GI cells, as these occur earlier in the embryonic development, but may be impacting their maturation. Although comparative studies between rat and humans are limited, Clancy et al. made a prediction of neuronal events model, which allow us to predict that E18 and E19 in rat corresponds to E67 and E92 in humans (10–13 weeks) [79–82]. This timepoint (E18 and E19) corresponds to the beginning of gut peristalsis in humans, which the presence of immunoreactivity to KIT is one of the peristalsis landmarks [74, 83].

In the present study, dysmotility was seen only in adult *iuGC* rats (3 and 8 months), after weaning, which may also reflect the importance of the microflora for postnatal maturation of ENS [84]. In fact, some studies showed a relation between different feeding periods with ENS development [85, 86] and not only can GC affect microflora environment [87–89] but, also, dysmotility itself can impact the distribution of intestinal microflora in the digestive tract [90, 91]. ICC, which generate slow waves responsible for peristalsis in the small intestine [78, 92], go through a differentiation process over the first month, from birth until weaning to adulthood phase [85]. Increased branching processes, as a network enteric maturation process, was found in colon’s mice over the first month in ICC-MP [93]. In addition, some studies in mice and guinea pigs, show that ICC continues proliferating until P24-35, despite decrease density [93–95].

*iuGC* treatment did not affect gut permeability. Although the role of GC in intestinal maturation, namely permeability, in humans (last trimester) and rodents (first weeks of life) has been described, little is known about the impact of prenatal stress in gut permeability [96–100]. After birth, rats go through a period of low levels of corticosterone (6–12) until day 14, when levels start to rise until reach a peak on day 24 [96, 101]. The increase of corticosterone levels are associated with enzymatic and mucosa cells maturation [101]. In the last years, studies have shown disruption of the intestinal barrier in rat gut under stress namely maternal separation (MS) [102, 103]. Accordingly with Moussaoui et al, corticosterone has a direct effect on gut permeability. Moussaoui et al showed that neonatal stress has impact colon permeability at day 10 but not at day 20 [102]. This seems to be related to GC’s sensibility, since when GC receptors are blocked MS rats display normal epithelial barrier function. Different from this MS model, *iuGC* animals show normal corticosterone levels [11], which may explain our results.

The GI tract has 95% of total 5-HT in the body [104] and all 5-HT in the blood is derived from the gut (90% of 5-HT is secreted by Enterochromaffin cells, EC) [37]. EC are present in the duodenum since E15 [105], when levels of 5-HT or enteric neural 5-HT receptors arise [106].We found low levels of 5-HT in the gut of *iuGC* animals, particularly at 3 months old. The administration of sGC (including DEX) has been shown to impact 5-HT levels, in the CNS, through trypyophan hydroxylases (THP1 and TPH2), the rate-limiting enzyme for biosynthesis of 5-HT [107, 108], also present in the EC. However, there are few studies approaching the impact of sGC, administered during pregnancy, in the TPH. Hiroi R. et al showed that *iuGC* increases the expression of TPH2 mRNA at P7, but decreases in adulthood, in the CNS (dorsal raphe nucleus) [109]. Interestingly, previous studies, showed that *iuGC* leads to a hyperanxious phenotype in adulthood [7, 41] at 2–3 months old, the time point which we are reporting decreased 5-HT levels in the gut. Moreover, *iuGC* may be impacting 5-HT levels through regulation of 5-HT receptors. Nagano et al found a decrease of 5-HT_1A_ receptor mRNA in medial prefrontal cortex (mPFC) and 5-HT content in the hippocampus of *iuGC* animals [110]. Both the mPFC and the hippocampus express GR and MR [111, 112] and have been shown to influence GI motility, which may link the changes observed in the brain and in the periphery [113–115]. It is known that distribution of 5-HT receptors occurs in a cephalocaudal way. During ontogeny the binding of 3H-5-HT appears for the first time on day E14, in the stomach and small intestine from the pylorus to the levels of the mid-jejunum. 3H-5-HT binding sites then spread distally, reaching the ileocolic sphincter on day E15, the proximal colon on days E-16-18, and the distal colon on day P2 [116]. As reviewed somewhere else, it has been suggested that stimulation of 5-HT2B receptors by 5-HT influences the fate of late-developing enteric neurons. In foetal mice at E14-E16 mRNA for 5-HT2B receptor was found in every ganglion of the developing myenteric plexus. At 18 the proportion of neurons expressing 5-HT2B receptor mRNA declined to the low adult levels. Moreover, activation of these receptors increases proliferation of ICC cultured from neonatal mouse jejunum [117]. Thus, administration of DEX at E18-19 may be impacting the expression of 5-HT2B in time which could reflect changes in serotonin levels in adulthood. This is a speculative hypothesis, yet to be explored. Although it has been shown changes in GI tract with age, little is known about the changes in serotonin levels with aging [118]. Our results add to the lacking literature in this topic. Taken all together, *iuGC* can impact 5-HT levels in the gut and within the CNS, which suggest a bidirectional communication between brain and gut.

## Conclusion

Although GC are widely administered to pregnant women, studies are still lacking regarding the impact of this administration on the GI tract [119]. Despite the great improvement in survival of preterm babies with prenatal DEX administration, little is still known on possible adverse effects of this administration in adulthood. The present study shows that *iuGC* differentially impacts GI motility, proliferation and apoptosis rate as well as serotonin levels. These effects are age-dependent.

## Supporting Information

S1 Fig Small intestine length normalized per weight at 24 hours and 1, 3 and 8 months old. The length of the small intestine was normalized per animal weight and no differences were found, n = 5, 6, 9, 6 CTR and n = 3, 9, 11, 7 *iuGC*. *iuGC*, *in utero* glucocorticoid exposed animals.
(TIF)

S2 Fig Impact of acute/chronic gavage on GI transit, at 3 months old. Acute gavage, as acute stressor, leads to a decrease in transit in Control Wistar rats. After 5 days of chronic gavage, while Control rats have normal transit, *iuGC* show dysmotility; n = 5 and 5 CTR and n = 4 and 4 *iuGC*. *iuGC*, in utero glucocorticoid exposed animals.* P <0.05
(TIF)

S1 File Minimal Data Set. The attached file contains the minimal dataset for the above study, including the data from (1) small intestine’s length, (2) layers’ length in the small intestine, (3) rate of apoptosis and mitosis in the small intestine, (4) upper gastrointestinal transit and permeability, (5) serotonin levels in the small intestine, (6) normalized data (length per weight) and (7) upper gastrointestinal transit after acute and chronic gavage.
(XLSX)
