# Supplementary figures and images for: The Impact of Prenatal Exposure to Dexamethasone on Gastrointestinal Function in Rats
Source: PLoS One. 2016 Sep 1;11(9):e0161750. doi: 10.1371/journal.pone.0161750 (PMC5008745; doi:10.1371/journal.pone.0161750)

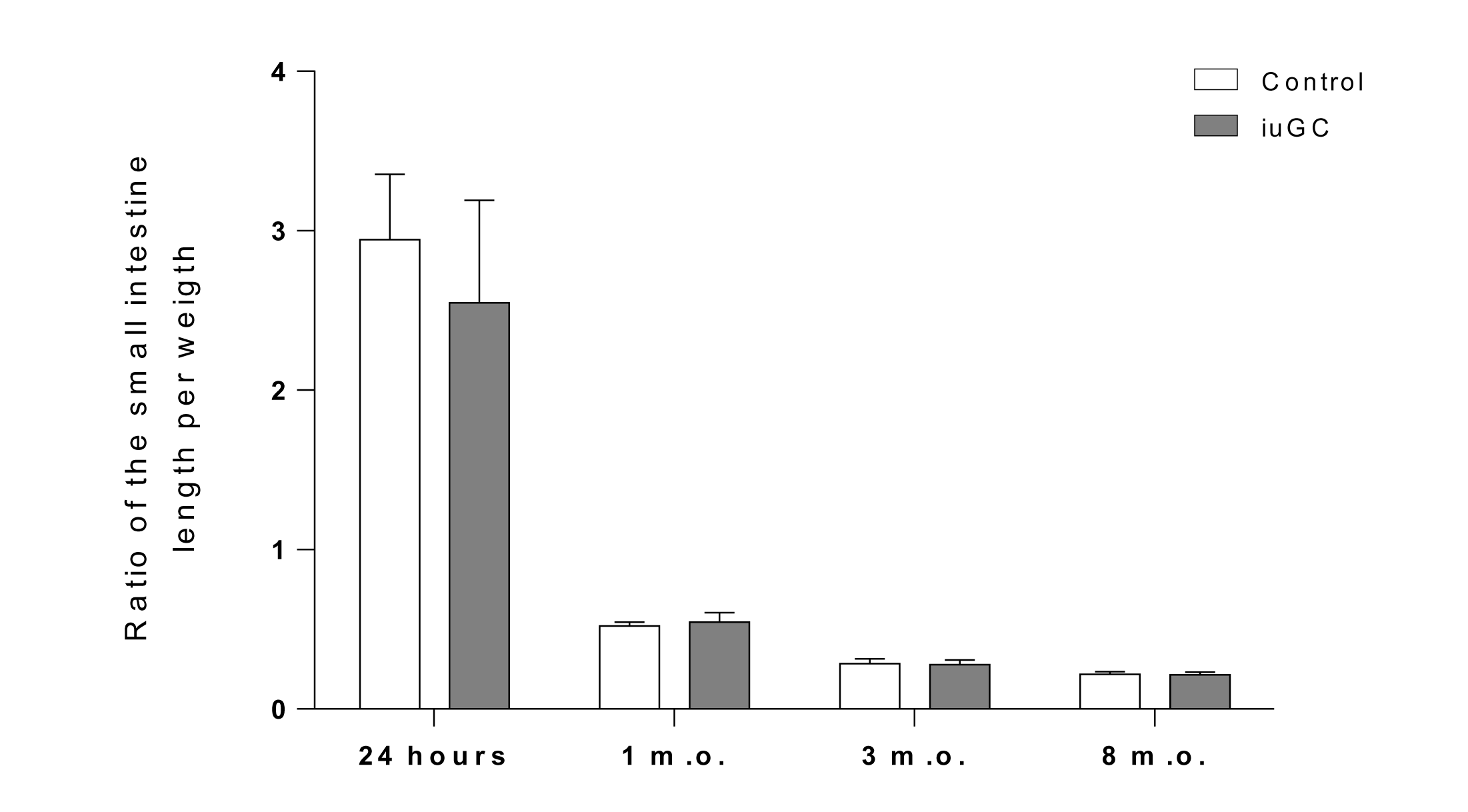

Supplement: S1 Fig — The length of the small intestine was normalized per animal weight and no differences were found, n = 5, 6, 9, 6 CTR and n = 3, 9, 11, 7 iuGC. iuGC, in utero glucocorticoid exposed animals. (TIF) [file pone.0161750.s001.tif]

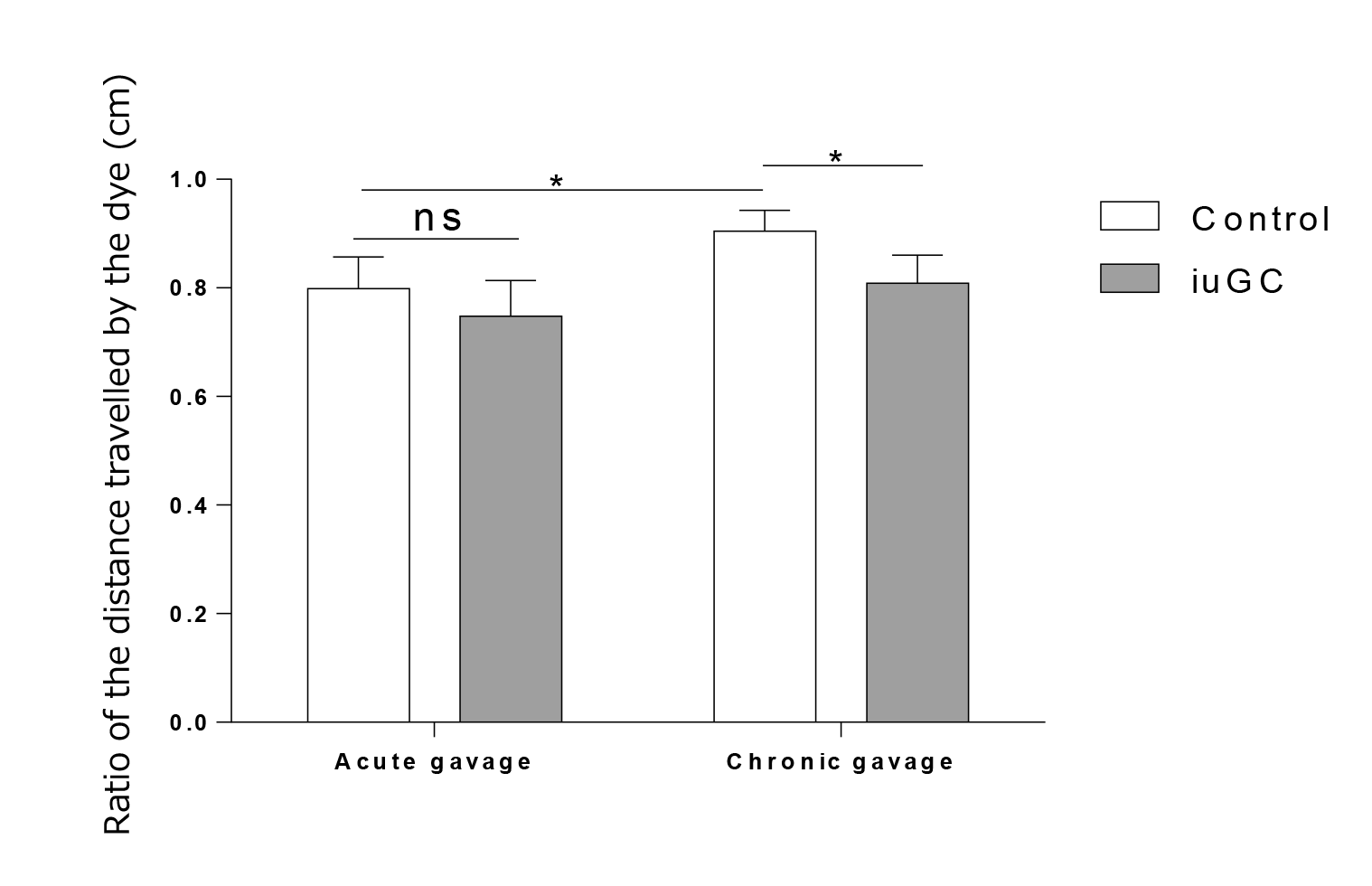

Supplement: S2 Fig — Acute gavage, as acute stressor, leads to a decrease in transit in Control Wistar rats. After 5 days of chronic gavage, while Control rats have normal transit, iuGC show dysmotility; n = 5 and 5 CTR and n = 4 and 4 iuGC. iuGC, in utero glucocorticoid exposed animals.* P <0.05 (TIF) [file pone.0161750.s002.tif]
